# Adolescents on Social Media: Aggression and Cyberbullying

**DOI:** 10.11621/pir.2021.0412

**Published:** 2021-07-12

**Authors:** Vladimir S. Sobkin, Aleksandra V. Fedotova

**Affiliations:** a Center for Sociology of Education, The Federal State Budget Scientific Institution Institute of Education Management of the Russian Academy of Education, Moscow, Russia; b Faculty of Psychology, Lomonosov Moscow State University, Moscow, Russia

**Keywords:** Social media, adolescence, aggression, cyberbullying, motives for using social media, self-presentation, activity on social media, intensity of the use of social media, gender speci cs, social status

## Abstract

**Background:**

The aggressiveness of social networking is a significant component of the risk modern teenagers face during socialization, and cyberbullying is one of the most controversial forms of aggressive behavior on social media.

**Objective:**

This paper deals with the study of secondary school students’ behavior on social media. The parameters characterizing teenagers’ usage of social media — their activity, intensity, motives, and self-presentation — are analyzed with respect to gender, age, and social psychological factors. The main focus is teenagers’ personal experience dealing with aggressive situations on social media: their role in aggressive situations (as aggressor, victim, or witness); the form of aggression (public or private); the aggressor’s characteristics (acquaintances or strangers, persons, or groups); and their views on what action victims should take (ignoring it, confronting it, or asking for help).

**Design:**

This article is based on data obtained by researchers at the Center for Sociology of Education of the Institute of Education Management of the Russian Academy of Education in 2020–2021. Using a specially developed questionnaire, we collected responses from 40,575 students from grades 7–11 in 17 regions of Russian Federation through an anonymous online survey. Mathematical statistical methods were used for data processing, specifically, the chi-square test in the “Basic statistics-Difference tests” module of the “StatSoft Statistica 7.0” package.

**Results:**

The data showed that the adolescents with high status among their classmates (“leaders”) used social media as an important educational resource, while those with low status (“loners”) used it to compensate for their poor real-life experience. Aggression on social media appears to be quite common among adolescents. The traditional differences between male and female subcultures appeared in the choice between private or public forms of aggression. The increase in aggressive interactions with strangers as the youth aged indicated that the realization of the teenage distinctive basic need for “expanding one’s social environment” in online interaction comes with the risks of encountering unfriendly, aggressive reactions.

**Conclusion:**

Communication on social media reflects an adolescent’s real-life interaction in school: those who have experienced psychological or physical bullying are more likely to become both victims and offenders in aggressive situations on social media. Th is transfer of group bullying from real life to the virtual can be seen as the main feature of adolescent cyberbullying.

## Introduction

Digital technologies these days play a huge role in all areas of human life. A person learns to use computers and smartphones in early childhood (Sobkin & Skobel’tsina, 2014), and adolescents, due to modern mobile devices, almost “live on the Internet” (Koroleva, 2016; Sobkin & Fedotova, 2019c). Thus social media is the space where crucial processes of adolescent maturation take place: the development of self-awareness, the creation of identity and self-determination, and the phenomenon of “expanding one’s social environment” (Bozhovich, 2008; El’konin, 1989; Vygotsky, 1984). Moreover, many modern researchers distinguish social networks as a special institution of socialization (Belinskaya, 2013; Koroleva, 2015; Martsinkovskaya, 2010; Sobkin & Fedotova, 2019b; Soldatova, 2018).

The popularity of social networks among adolescents is a matter of concern for the pedagogical and parental communities, and increased interest among researchers. Initially the networks were considered mainly from the point of view of their potential risks (Brenner, 1997; Karabanova & Molchanov, 2018; Marino, Gini, Vieno, & Spada, 2018; Shuktueva, 2016; Soldatova & L’vova, 2018; Voiskounsky, 2010). After various crises (teenage suicides, shooting in educational institutions, etc.), some publications would always blame the influence of social networking (Korolenko, Dmitrieva, & Levina, 2014; Mursalieva, 2016; Soldatova & Iliukhina, 2021). Therefore, the study of teenage aggression on social networks is extremely valuable.

In speaking about adolescents and aggression on social media, we would like to clarify that we are facing two different phenomena. On the one hand, we are talking about the adolescent’s perception of aggression that is not directly related to him; this is one kind of aggressive content on social networks, including socially acceptable forms (news, movies, sports events, etc.). On the other hand, there is the aggressive behavior of social network users, directed at other participants in the network interaction, in particular at the respondent himself (threats, insults, harassment, etc.). Cyberbullying, which today is the object of close attention of researchers, has a special place here (Beale & Hall; 2007; Bochaver & Khlomov, 2014; Fanti, Demetriou, & Hawa, 2012; Griezel, Finger, Bodkin-Andrews, Craven, & Yeung, 2012; Hinduja & Patchin, 2010; Kowalski & Limber, 2013; Li, 2010; Menesini & Salmivalli, 2017; Raskauskas & Stoltz, 2007; Smith, Mahdavi, Carvalho, Fisher, Russel, & Tippett, 2008; Song & Oh, 2018; Walrave & Heirman, 2011; Williams, Cheung, & Choi, 2000).

The present study originated in the early 2000s when we started our research on the characteristics of the information environment of schoolchildren (Sobkin & Evstigneeva, 2001). In our previous publications we have considered such aspects of adolescents’ interaction on social networks as the motivation and content of communication, the risks and consequences of using social networks, social network users’ psychological well-being, and their attitudes toward aggression on social media (Sobkin & Fedotova, 2018a, 2018b, 2019a, 2019b, 2019c; Sobkin, 2016). In this paper we focus on the adolescents’ personal experiences of participation in aggressive situations on the social network: their actual role in those situations (aggressor, victim, or witness); the forms of aggression (public or private); the bully’s characteristics (acquaintances or strangers, persons or groups); and their views on how the victim should respond during the situation of cyberbullying (ignoring, confronting, or asking for help).

## Methods

This article is based on data obtained during an anonymous electronic questionnaire survey, which was conducted in 2020-2021 by the research group of the Center for Sociology of Education of the Institute for Education Management of the Russian Academy of Education. The opinions of 40,575 schoolchildren (40.2% males, 59.8% females) in grades 7 (24.7%), 8 (23.3%), 9 (22.0%), 10 (16.0%), and 11 (14.0%) from 17 regions of the Russian Federation (19.6% from regional centers, 13.6% from district centers, 31.9% from small towns, 35.0% from rural areas) were received.

The special questionnaire was developed to study various aspects of adolescents’ deviant behavior. It contained more than 150 open, closed, and scaled questions. The questionnaire featured questions concerning the teenagers’ behavior on social media and their socio-demographic characteristics. The answers were processed by SPSS 21 and StatSoft Statistica 7.0. To compare the proportions observed in two independent samples and expressed as percentages, the chi-square test in the “Basic statistics— Difference tests” module of the StatSoft Statistica 7.0 package was used. All the differences indicated in the article are statistically significant at the 0.05 level.

When processing the empirical data, we paid special attention to the social and role aspects of the interaction in the aggressive situations, the forms of aggression, and the characteristics of the subjects of aggression, as well as the opinions of the adolescents about how a victim of aggression in social networks should respond. The data obtained were analyzed in relation to the influence of three groups of factors: demographics (gender, age); the particularities of their use of social networks (activity, intensity); and their social and psychological well-being (status among classmates, academic success, and real-life bullying experience).

## Results and discussion

Our data are grouped into two sections. The first presents the characteristics of the adolescents’ use of social networks (activity, intensity, motivation, and self-presentation). The second section examines the experience of a teenager’s encounter with aggressive situations on social media: their role (aggressor, victim, or witness); the form of aggression; the characteristics of the subject of aggression; and possible reactions of the victim to the aggression.

### 1. Characteristics of adolescents’ behavior on social networks

This section contains a general description of the adolescents’ interaction on social networks. It examines how actively adolescents used social networks, the particularities of their motivation to use the network, and their self-presentation on the network.

*Activity and intensity*. In the course of the survey, only 2.3% of respondents indicated that they do not use social networks. Thus, online communication is already an everyday reality for modern schoolchildren. At the same time, questions arose about the activity and intensity of network communication.

The answer to the question about the teens’ activity on social networks required the adolescents’ self-assessment of their involvement on the social network (how much they monitor their account, make changes, etc.). Almost half of the respondents (48.6%) noted that they are “moderate users,” indicating that they update their page from time to time and communicate with friends. About a third of adolescents (30.8%) identified themselves as “active users:” they constantly monitored their account, updated it, participated in active correspondence, and looked for new friends. A tenth (13.3%) noted that they used social networks “from time to time,” rarely updated their pages, and only visited it as needed. And only a few (5.0%) indicated that they rarely used social networks and had never updated their page.

**Figure 1.**
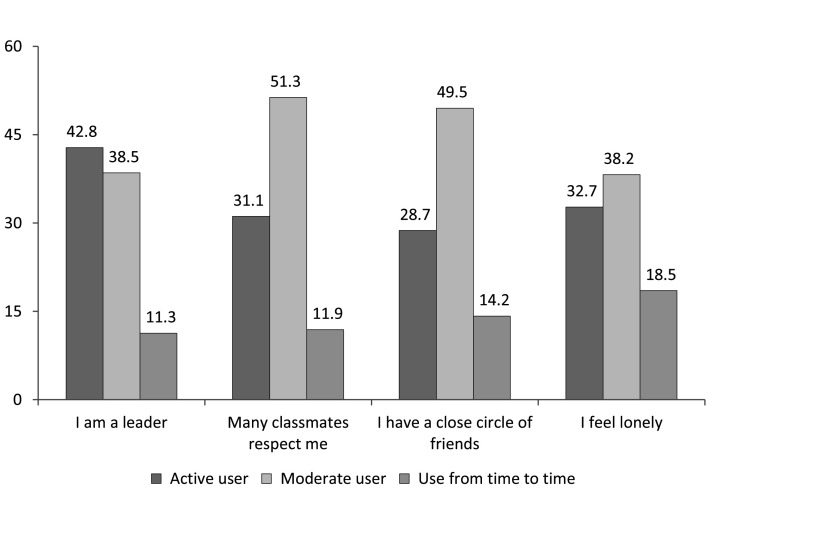
Adolescents’ activity on social media depending on their social status among classmates (%)

When analyzing the data, we paid particular attention to the connection between the activity of using social networks and the adolescent’s self-assessment of his status among his or her classmates (*Figure 1*).

As is shown in the figure, the highest proportion of active users was among those adolescents who considered themselves “leaders;” students with “average status” were more likely to identify themselves as moderate users; “loners” were more likely to indicate that they used social networks from time to time. These data show that network activity reflects the distribution of status in the adolescents’ real interpersonal communication. In this respect, the online environment is a significant social space, and teenage “leaders” aspire to take over this information field and establish themselves in it.

Responses on the *intensity* of using social networks (the time that adolescents devote to social networks during the day) showed that more than a third of students (35.2%) devoted 1-3 hours daily to this activity. About a quarter spent 5 or more hours (25.7%) and from 3 to 5 hours (25.6%) on social networks. Only 13.5% of respondents spent less than an hour a day on social networking. It is worth noting that the proportion of such answers was significantly higher among boys: 21.3% spent less than an hour on the networks, while among girls it was just 8.2%.

The share of schoolchildren who intensively communicated on social networks (from 3 to 5 hours or more) consistently increased during their time in secondary school: among seventh graders, the figure was 45.3%, and by the 9th grade, it was already 54.6%. However, by the end of school, the intensity of network communication was somewhat reduced (to 52.0%).

It should be specially noted that adolescents with low personal status among the classmates (feel “lonely”) were more likely than others to spend more than 5 hours on social networks (36.0%). The same tendency manifested itself among those schoolchildren who in real life participated in fights and were subjected to psychological and physical violence. This leads to the conclusion that communication on social networks performs a certain socio-compensatory function for a significant number of adolescents. In this regard, a large amount of time spent on network communication (more than 5 hours per day) is typical for those adolescents who assess their personal status in the class as low, as well as those participating in fights in real life and those subjected to psychological and physical violence (bullying).

#### Motivation

The impact of social networks was largely determined by the goals and motives which guided the adolescents in using them. During the survey, the respondents were asked about their motivation for the use of social networks. The distribution of answers is shown in *Table 1*.

**Table 1:** Motives for using social networks (%)

	Average	Male	Female
To have fun	47.5	51.8	44.6
To improve educational level	23.8	22.7	24.7
To master new skills	17.7	18.3	17.3
To receive the necessary information	44.5	42.1	46.6
Boredom	43.6	44.7	43.1
Conflicts and difficulties in real life	7.8	5.2	9.4
The possibility to freely express your point of view	11.9	10.8	12.6
To communicate	61.9	56.4	66.2
The opportunity to make money	7.1	8.6	6.1
To meet new people	24.1	20.6	26.4
The ability to do things that are not available in real life (online excursions, communication with foreigners, etc.)	9.4	7.6	10.8
To keep abreast of events	42.3	37.4	46.0

Comparative analysis of gender differences (see *Table 1*) showed that boys were more likely than girls to be guided by “the desire to have fun,” “the desire to master new skills,” and “opportunities to make money.” Girls, on the other hand, more often pointed to such motives as “the desire to communicate,” “to receive information,” and “the desire to keep abreast of events,” “to meet new people,” and “to escape conflicts in real life.”

It should be noted that, with age, the importance of the informational and educational motives increased, and the frequency of choosing such a motive as “boredom” decreased. This indicates that by the time of graduation, social networks more often appeared as information resources, and not just as a way to “kill time.”

As expected, the schoolchildren with high academic performance were more focused on educational motives, and the desire to communicate and realize what is not available in real life. Low-performing students were more likely to be driven by boredom, a desire to make new acquaintances, and a desire to earn money.

In the context of this article, the analysis of the influence of the adolescent’s assessment of his personal status in the class on his motivation for social networking was of particular interest. Thus, schoolchildren with a low social status (those who “feel lonely” and those whose “social circle is limited”) more often noted that they turned to social networks “out of boredom” (respectively: 53.5% and 51.5%) than their more popular peers did (37.7% among those who were “respected” and 33.5% among “leaders”).

Typically, it was conflicts in real life that became the reason for turning to social networks among those adolescents who felt lonely in their class: 23.6% gave that reason (among those whom “many respect,” the percentage was 4.8%; among the “leaders,” 5.8%). “Lonely” adolescents also more often used social networks for the opportunity to express their point of view: 18.2%, compared to 10.3% among the “respected,” and 10.8% among the “leaders”. In addition, “loners” were more interested in the possibilities of social networks that were associated with “realizing goals that are not available in real life:” 14.3%, compared with 10.6% among “leaders” and 9.0% among “respected.”

Finally, it should be noted that the adolescents who identified themselves as leaders were more likely to view social media as an educational resource. They were more often guided by “the desire to improve their educational level”— 31.4%, compared with 21.3% among adolescents with a lower social status— and the desire to master new skills— 21.3%, compared with 16.7% among “having a limited circle of friends.”

Th us, our data gave support for identifying two different orientations: 1) schoolchildren with a low self-esteem and personal status among classmates seeing social networks as a way to compensate for negative trends in real life and overcome the barriers to self-realization (overcoming boredom, avoiding conflicts, the ability to express their point of view); and 2) then socially successful schoolchildren using the capabilities of social networks as an educational resource.

#### Self-presentation

One of the important features of social networks is the relative freedom to choose a behavioral pattern and present oneself in the space of network communication. In this regard, the adolescents were asked to characterize their page on the social network from the point of view of its perception by other users, choosing among several proposed options.

The overwhelming majority (82.8%) classified their page as “ordinary.”

At the same time, the additional characteristics used by the respondents were also important, since self-presentation on the network makes it possible to clarify the target of the adolescent’s communication. Thus, about a third (29.7%) noted that their page was “interesting only for friends,” and a quarter of the respondents (24.7%) described their page as “reserved.” Only 8.4% of the schoolchildren thought that their page might be of interest to strangers; 7.3% thought their page was “useful;” 5.3% considered their page to be “extraordinary;” 3.1%, “expansive;” and 1.7% “provocative.”

Note that the distribution of the answers was grouped around two contrasting orientations. One of them was “friends vs. strangers.” Here we can see that, in general, the schoolchildren had a predominant orientation toward maintaining communication with a close circle (“friends”). Moreover, this was typical for a third of the respondents. At the same time, almost one in five was clearly focused on contact with strangers, trying to “attract” or “interest” them. Here another tendency characteristic of adolescence was manifested— “the expansion of the social environment.” Another contrast concerned emotional manifestations: “reserved vs. expansive.” If every fourth teen was inclined to be quite reserved when communicating on the network, then every tenth, on the contrary, was focused on presenting himself in situations of network communication as “extraordinary,” “expansive,” or “provocative.”

A special analysis of our data on the activity and intensity of using the network showed that adolescents who considered themselves to be active users more often noted that their profile was attractive to both friends and strangers, and that it was “useful.” Moreover, active users more often, compared to those who pay less attention to social networks, classified their page as “extraordinary,” “expansive,” and “provocative.” The same tendencies were also characteristic of the intensity of Internet use: adolescents who spent more time on the social networks tended to classify their page as “expansive,” “extraordinary,” “provocative,” or “attractive to unfamiliar users.”

It is important to pay attention to the fact that adolescents with both high and low status among classmates, more often than adolescents with an average status, characterized their page as “expansive,” “extraordinary,” and “provocative.” At the same time, in contrast to students with a low status among classmates, leaders more often classified their page as “useful.” Of fundamental importance in the context of this article was the fact that adolescents who had been bullied in real life were much more likely to refer to their page as “expansive,” “extraordinary,” and “provocative.” This leads to the conclusion that the communication on social networks plays a compensatory role for a significant number of students subjected to school bullying.

### 2. The experience of facing aggression and cyberbullying on social networks

This section is devoted to the adolescents’ experience of direct encounters with aggressive situations on social networks. Here we will consider the points related to the role of the teenager (“aggressor”, “victim”, or “witness”) in such situations; the forms of aggression (“privacy vs. publicity”); the characteristics of the subject of the aggression (“familiar vs. unfamiliar”); and opinions on how a victim of aggression should behave on social networks (ignore aggression, confront it, or seek help).

#### Role in the aggressive situation

Slightly less than half of the surveyed schoolchildren (43.8%) indicated that they did not encounter interpersonal aggression on social networks. For the rest, aggression on social networks was a fairly widespread phenomenon: every fourth person (24.4%) faced “aggression towards themselves from other users;” almost the same number (26.6%) indicated that they “witnessed aggression against other users;” relatively few (5.2%) admitted that “they themselves acted as an aggressor against other users.”

Analysis of gender specifics showed that network interaction among boys was more aggressive: they more often noted that they had had an experience of being both the aggressor (7.0% for boys vs. 4.0% for girls) and the victim (respectively: 26.6% for boys and 22.8% for girls). Girls more often indicated that they had not encountered interpersonal aggression on social networks (46.3% vs. 40.1% for boys).

The study of age dynamics showed an increase in the aggressiveness on social networks over the course of education in junior high school. This was evidenced by the decrease in the proportion of students who had never encountered aggression: 47.1% in the 7th grade and 41.4% in the 9th grade. This was mainly due to an increase in the proportion of “witnesses” of network aggression from the 7th to the 9th grade (respectively: 21.9% and 26.9%).

The academic success of a teenager also affected the level of aggressiveness of his network environment. Thus, among the “C-graders,” the share of “victims” of aggression (26.9% vs. 21.2% for “A-graders”) and “aggressors” (7.1% vs. 3.6% for “ A-graders”) was somewhat higher than among “A-graders.” Thus, low academic success was generally associated with situations of increased aggressive behavior on social networks. This coheres with the results of Kowalski & Limber’s research, which stated that adolescents in the cyber bully/victim group had the most negative scores on most measures of psychological health, physical health, and academic performance (Kowalski & Limber, 2013).

The most significant differences related to the adolescents’ assessment of their status among classmates. Popular adolescents more often indicated that they had not had to deal with cases of aggression in social networks (46.9% among the “leaders,” compared to 30.7% among the “loners”). Moreover, among the “loners,” 38.6% noted that they had been objects of aggression from other network users, but among the “respected” students, this number was almost two times less— 20.7%.

**Figure 2.**
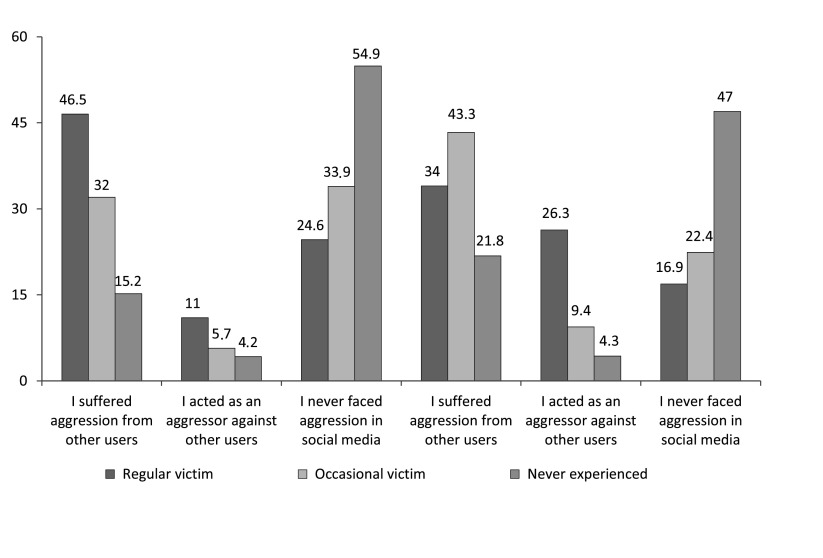
Adolescents’ encounter with aggressive situations on social media depending on their real-life bullying experience (%)

These data give grounds for the assumption that adolescent aggression in real life is carried over to situations of network communication: the interpersonal status of a teenager in school also affected the attitude toward him or her in network communication. For this purpose, we carried out a special analysis to compare the frequency of experiencing psychological or physical violence (bullying) in real life and the role of the adolescent in network aggression (victim, aggressor, or witness) (see *Figure 2*).

The data presented in the figure show that teenager’s experience of psychological or physical bullying increased the likelihood of his or her participation in situations of network aggression in the role of both the victim and the offender. In this regard, our results correspond with those of prior studies (Raskauskas & Stoltz, 2007; Smith, Mahdavi, Carvalho, Fisher, Russel, & Tippett, 2008; Fanti, Demetriou, & Hawa, 2012; Kowalski & Limber, 2013), which showed that the traditional victims and bullies are likely to retain their roles across the contexts of school and the cyber world. However, in those studies the possibility that adolescents who are victimized at school would become electronic bullies was not supported, whereas our data clearly shows the higher rates of cyber aggressors among those who had suffered from psychological and especially physical bullying at school.

#### Form of aggression

In order to clarify the features of network aggression, those adolescents who indicated that they were “victims” of aggression were asked to specify in what form it was expressed. The majority (55.0%) answered that they received personal messages containing insults, ridicule, harassment, threats, etc. More than a third of respondents (36.5%) indicated that the aggression consisted in public “showdowns” with other users (“holy wars,” “flaming,” “hating,” “trolling,” etc.); 8.5% noted that the aggression was expressed in public posts, containing insults, mockery, harassment, threats, etc.

Boys more often indicated that the aggression was expressed in public confrontations (41.9% compared to 32.2% for girls); girls, on the other hand, more often noted that the aggression was manifested in private messages (60.2% compared to 48.4% for boys). In our opinion, such results are a consequence of traditional differences in the forms of aggressive behavior between the male and female subcultures: among boys we more often observe open public confrontations (“duels”), while for girls, individual-personal (“hidden”) aggression is more common.

It should be noted that the answers about the forms of manifestation of aggression in the network did not vary with the socio-demographic and sociocultural factors. At the same time, it is noteworthy that adolescents with low academic performance and low interpersonal status in the classroom more often noted that aggression expressed towards them came in the form of public posts; and those who had suffered psychological and physical bullying in real life more often indicated the public nature of the network aggression. In general, this allows us to conclude that the space of network communication turns out to be an important medium of very public pressure on the individual.

#### Subject of aggression

Another aspect of aggressive behavior on social media concerns the characteristics of the aggressor in relation to the parameter of his anonymity (more precisely: “familiar vs. unfamiliar”). Most of the adolescents who had been subjected to aggression indicated that the aggression came from unfamiliar users (63.7%); almost every fifth teen (17.8%) noted that it was someone from their real contacts; every tenth (11.9%) became a victim of aggression from a group of unfamiliar users; and 6.6% were subjected to aggression by a group of real acquaintances. Thus, three quarters of those subjected to aggression on the network indicated that it came from strangers.

Gender analysis showed that boys more often than girls faced aggression from unknown users (respectively: 69.7% and 59.1%) or a group of unknown users (respectively: 13.1% and 10.9%). Girls, on the other hand, more often pointed to aggression on social networks from their real acquaintances— 21.5% (it is 13.2% for boys)— or a group of real acquaintances (8.5% and 4.0%, respectively).

The proportion of strangers among the aggressors increased with the students’ age (from 63.4% in the 7th grade to 68.8% in the 11th grade), and the cases of aggression from real acquaintances decreased (respectively: 17.1% and 13.8 %). In principle, our data indicated that the general age tendency associated with the “expansion of the social environment” of the adolescent, in situations of network communication, was often associated with the risk of aggression on the part of strangers.

**Figure 3.**
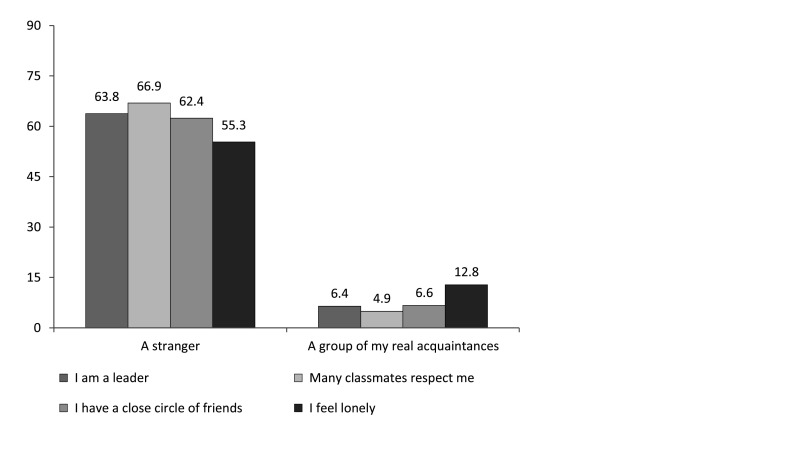
Aggressor’s specifics depending on adolescents’ social status among classmates (%)

In this regard, the manifestation of aggression on the network on the part of “acquaintances / strangers” in relation to the adolescents’ different interpersonal status among their classmates is of particular interest (see *Figure 3*).

Students who “feel lonely,” compared to their more popular peers, were less likely to indicate that they experienced aggression from strangers, but more often that they had been the victim of aggression from a group of real acquaintances. It can be assumed that in this case, we are dealing with cyberbullying, when bullying moves from the real to the virtual space, keeping the same roles for the parties to the conflict.

#### Victim reaction

An important aspect of behavior that relates specifically to adolescent cyberbullying is the definition of how a victim should behave in such a situation: ignore aggression, respond in the same way, or seek help.

About a third of respondents (31.3%) believed that aggression on social networks should be ignored. One fifth (20.6%) considered it necessary to complain to the administration of the social network; 16.1% pointed to the need to tell their parents about it; 12.9% believed that it was necessary to inform the police. One tenth of adolescents (8.2%) thought that this should be shared with friends; 6.3% believed that they should respond in kind; 4.7% believed that in such a situation, it was necessary to turn to teachers for help. Comparing these with the results of previous research (Smith, Mahdavi, Carvalho, Fisher, Russel, & Tippett, 2008), we can see that the most popular strategies pupils advocated for cyberbullying was still avoidance.

Boys more often than girls, answered that such aggression should be ignored (respectively: 59.5% and 49.1%) or be responded to in kind (respectively: 13.7% and 8.6%). Girls, in turn, more often tended to seek help from the network administration (respectively: 39.1% and 29.2%), their parents (respectively: 34.7% and 16.2%), or the police (respectively: 26.1% and 15.5%).

Throughout secondary school, the students were more often focused on responding to aggression on social networks in kind, or telling teachers about its manifestations. By the time they graduated from school, they were more likely to seek help from the network administration (40.6% in the 11th grade compared to 29.4% in the 7th grade) or from the police (25.6% and 19.9%, respectively).

Students with a lower level of academic performance were more likely to respond with aggression to aggression (12.9% among C students, 9.5% among B students, 8.8% among A students), while students who demonstrated a high level of academic performance were more focused on notifying the network administration (41.2% among excellent students, 37.9% among B students, and 29.3% among C students), parents (34.2%, 29.4%, and 22.2%, respectively), and friends ( respectively: 16.3%, 14.5%, and 12.1%).

Teens who had participated in fights during the past two months were more likely to respond that aggression on social media should be ignored (54.7% versus 51.0% among those who do not fight) or responded to in kind (respectively: 20.6% vs. 7.6%). Those who had never fought were inclined to resolve the situation of the aggression towards them in by involving their parents (32.3% compared to 18.4% among those who had participated in fights in the last two months), the network administration (respectively: 39.6 % vs. 25.8%), and the police (respectively: 24.9% vs. 16.5%).

Finally, victims of regular bullying were less likely to focus on turning to the police in case of network aggression than those who were bullied occasionally or never (19.0% of victims of regular psychological bullying versus 21.7% and 22.4%; 14.2% among victims of physical bullying, compared with 19.6% and 22.4%, respectively). Teens who experienced bullying were more likely to point out that they should respond in kind to online bullying. Those who had been regularly exposed to aggression were less focused on telling their parents about what happened or informing the police.

In general, the above data show that the choice of a method of responding to aggression in a situation of network interaction is largely determined both by the influence of gender and age factors, and by the experience of resolving conflict situations in situations of real interaction.

## Conclusion

The analysis of our results showed that social networks play an important role in the daily life of a modern Russian teenager. Summarizing the results of the study, we highlight the following main conclusions:

1. The parameters characterizing the characteristics of adolescents’ involvement in network communication (activity and intensity of use, motivation, self-presentation) are largely determined by gender and age, as well as socio-psychological factors (academic success and self-assessed status among classmates).
2. The activity and intensity of adolescents’ behavior on social networks are correlated with their positions in the hierarchy of status in their real interpersonal communication. Moreover, there are two different orientations: when socially successful, schoolchildren use the possibilities of the social network as an important educational resource; but for schoolchildren with a low status in school, social networks perform an important compensatory function. This tendency is especially pronounced among a significant number of students who are subjected to school bullying.
3. The manifestation of aggression on social networks is quite common. At the same time, traditional differences in the forms of aggressive behavior in male and female subcultures are found here: if boys more often point to the public nature of network aggression, then girls tend to express aggression in the form of private messages. Of interest is the age-related dynamics of manifestations of network aggression in the context of general psychological ideas about the tendency of “expanding the social environment” in adolescence. The increase in aggressive manifestations on the part of unfamiliar network participants as the teens age indicates that the realization of the basic age-related need for “expanding the social environment” in situations of network comunicatimon is associated with the risks of encountering unfriendly, aggressive reactions.
4. The social status of a teenager among his or her classmates also significantly affects his or her encounters with aggression during network interaction. It has been established that manifestations of aggression in real life are carried over to situations of network communication, resulting in the same roles for the parties to the conflict. A teenager’s experience of psychological or physical bullying increases the likelihood of his participation in situations of network aggression in the role of both victim and offender. In this regard, it is quite indicative that adolescents who “feel lonely among their classmates” more often report aggression towards themselves from a group of real acquaintances. These features characterize adolescent cyberbullying, when group bullying moves from real to virtual space, where it becomes public.

## Limitations

The limitation of this research was the number of analyzed factors. We haven’t yet explored the connection between adolescent aggression on social media and their deviant behavior, as well as value orientations. Furthermore, we didn`t analyze the correlation between teenagers’ aggression on social networks with their extremist attitudes. We expect aggressive behavior to be a part of a larger set of attitudes.

Based on the data we presented in this work, we feel that closer study of adolescents who are in the at-risk group— those with low status among classmates and those who have had bullying experience— would be extremely useful for predicting, and possibly preventing, teenage cyber aggression.

Also, an expansion of the age span of the sample would provide a more thorough study of the origin and development of teenager aggression on social media.

## References

[ref1] Beale, A.V., & Hall, K.R. (2007). Cyberbullying: What School Administrators (and Parents) Can Do. The Clearing House: A Journal of Educational Strategies, Issues and Ideas, 81(1), 8–12.

[ref2] Belinskaia, E.P. (2013). Informatsionnaia sotsializatsiia podrostkov: opyt pol’zovaniia sotsial’nymi setiami i psikhologicheskoe blagopoluchie [Informational socialization of teenagers: the experience of using social networks and psychological well-being]. Psikhologicheskie issledovaniia [Psychological Studies], 6(30). http://psystudy.ru/index.php/num/2013v6n30/858-belinskaya30.html

[ref3] Bochaver, A.A., & Khlomov, K.D. (2014). Kiberbulling: travlia v prostranstve sovremennykh tekhnologii [Cyberbullying: Harassment in the space of modern technologies]. Psihologiia. Zhurnal Vysshei shkoly ekonomiki [Psychology. Journal of the Higher School of Economics], 11(3), 178–191.

[ref4] Bozhovich, L.I. (2008). Lichnost’ i ee formirovanie v detskom vozraste [Personality and its formation in childhood]. Piter.

[ref5] Brenner, V. (1997). Psychology of Computer Use: XLVII. Parameters of Internet Use, abuse, and addiction: The first 90 days of the Internet Usage Survey. Psychological Reports, 80(3), 879–882. 10.2466/pr0.1997.80.3.879 9198388

[ref6] El’konin, D.B. (1989). Izbrannye psikhologicheskie trudy [Selected psychological works]. Pedagogika.

[ref7] Fanti, K.A., Demetriou, A.G., & Hawa, V.V. (2012). A longitudinal study of cyberbullying: Examining risk and protective factors. European Journal of Developmental Psychology. Issue 2: Cyberbullying: Development, consequences, risk and protective factors, 9, 168–181.

[ref8] Griezel, L., Finger, L.R., Bodkin-Andrews, G.H., Craven, R.G., & Yeung, A.S. (2012). Uncovering the structure of gender and developmental differences in cyber bullying. The Journal of Educational Research, 105(6), 442–455. 10.1080/00220671.2011.629692

[ref9] Hinduja, S., & Patchin, J.W. (2010). Bullying, Cyberbullying, and Suicide. Archives of Suicide Research, 14(3), 206–221. 20658375 10.1080/13811118.2010.494133

[ref10] Karabanova, O.A., & Molchanov, S.V. (2018). Riski negativnogo vozdeistviia informatsionnoi produktsii na psikhicheskoe razvitie i povedenie detei i podrostkov [Risks of information products’ negative impact on children’s and adolescents’ mental development]. Nacional’nyj psihologicheskij zhurnal [National Psychological Journal], 3(31), 37–46.

[ref11] Korolenko, Ts.P., Dmitrieva, N.V., & Levina, L.V. (2014). K voprosu o vliianii Interneta na suitsidal’noe povedenie [On the issue of the Internet’s impact on suicidal behavior]. Uchenye zapiski SPbGIPSR [Th e Scientific Notes of St. Petersburg State Institute of Psychology and Social Work], 22(2), 103–107.

[ref12] Koroleva, D.O. (2015). Ispol’zovanie sotsial’nykh setei v obrazovanii i sotsializatsii podrostka: analiticheskii obzor empiricheskikh issledovanii (mezhdunarodnyi opyt) [The use of social networks in education and socialization of teenagers: an analytical review of empirical studies (international experience)]. Psikhologicheskaia nauka i obrazovanie [Psychological Science and Education], 20(1), 28–37. 10.17759/pse.2015200104

[ref13] Koroleva, D.O. (2016). Vsegda onlain: ispol’zovanie mobil’nykh tekhnologii i sotsial’nykh setei sovremennymi podrostkami doma iv shkole [Always online: modern teenagers’ using mobile technologies at home and in school]. Voprosy obrazovaniia [Issues on Education], 1, 205–224.

[ref14] Kowalski, R.M., & Limber, S.P. (2013). Psychological, Physical, and Academic Correlates of Cyberbullying and Traditional Bullying. Journal of Teenager Health, 53, 13–20. 10.1016/j.jadohealth.2012.09.018 23790195

[ref15] Li, Q. (2010). Cyberbullying in High Schools: A Study of Students’ Behaviors and Beliefs about This New Phenomenon. Journal of Aggression, Maltreatment & Trauma, 4(19), 372–392.

[ref16] Marino, C., Gini, G., Vieno, A., & Spada, M.M. (2018). The associations between problematic Facebook use, psychological distress and well-being among adolescents and young adults: A systematic review and meta-analysis. Journal of Affective Disorders, 226, 274–281. 10.1016/j.jad.2017.10.007 29024900

[ref17] Martsinkovskaia, T.D. (2010). Informatsionnoe prostranstvo kak factor sotsializatsii sovremennykh podrostkov [Information space as a factor of modern teenagers’ socialization]. Mir Psikhologii [The World of Psychology], 3, 90–102.

[ref18] Menesini, E., & Salmivalli, C. (2017). Bullying in schools: the state of knowledge and effective interventions. Psychology, Health & Medicine. Issue sup1: Know Violence in Childhood Global Learning Initiative: Dedicated to the memory of Peter Bell, 22, 240–253. 10.1080/13548506.2017.1279740 28114811

[ref19] Mursalieva, G. (2016). Gruppy smerti [The death groups]. Novaia gazeta [New Paper], 51, 2–5.

[ref20] Raskauskas, J., & Stoltz, A.D. (2007). Involvement in Traditional and Electronic Bullying Among Teenagers. Developmental Psychology, 43(3), 564–575. 17484571 10.1037/0012-1649.43.3.564

[ref21] Shuktueva, A.S. (2016). Sotsial’nye seti kak instrument dlia propagandy ekstremizma sredi podrostkov [Social networks as a media for extremism propaganda among adolescents]. Novoe slovo v nauke i praktike: gipotezy i aprobatsiia rezul’tatov issledovanii [New word in science and practice: hypotheses and approbation of research results], 25, 45–48.

[ref22] Smith, P.K., Mahdavi, J., Carvalho, M., Fisher, S., Russel, S., & Tippett, N. (2008). Cyber bullying: Its nature and impact in secondary school pupils. Journal of Child Psychology and Psychiatry, 49, 376–385. 18363945 10.1111/j.1469-7610.2007.01846.x

[ref23] Song, J., & Oh, I. (2018). Factors influencing bystanders’ behavioral reactions in cyberbullying situations. Computers in Human Behavior, 78, 273–282.

[ref24] Sobkin, V.S., & Evstigneeva, Yu.M. (2001). Podrostok: virtual’nost’ i sotsial’naia real’nost’. Po materialam sotsiologicheskogo issledovaniia. Trudy po sotsiologii obrazovaniia, VI, X. [Teenager: Virtual and social reality. Based on sociological survey. Works on the sociology of education, VI (10)]. Institut sotsiologii obrazovaniia RAO.

[ref25] Sobkin, V.S., & Fedotova, A.V. (2019a). Podrostkovaia agressiia v sotsial’nykh setyakh: vospriyatie i lichnyi opyt [Adolescent aggression in social media: perception and personal experience]. Psikhologicheskaia nauka i obrazovanie [Psychological Science and Education], 24(2), 5–18. 10.17759/pse.2019240201

[ref26] Sobkin, V.S., & Fedotova, A.V. (2018a). Podrostok v sotsial’nykh setiakh: k voprosu o sotsial’no-psikhologicheskom samochuvstvii [Teenager in social networks: on the issue of social and psychological well-being]. Natsional’nyi psikhologicheskii zhurnal [National Psychological Journal], 3(31), 23–36. 10.11621/npj.2018.0303

[ref27] Sobkin, V.S., & Fedotova, A.V. (2018b). Podrostok v sotsial’nykh setiakh: risk i reaktsii [Teenager and social networking: risks and response]. Voprosy psikhicheskogo zdorov’ia detei i podrostkov [Issues of Mental Health of Children and Teenagers], 1, 47–56.

[ref28] Sobkin, V.S., & Fedotova, A.V. (2019b). Set’ kak prostranstvo sotsializatsii sovremennogo podrostka [Social Media as a Field of a Modern Teenager’s Socialization]. Konsul’tativnaia psikhologiia i psikhoterapiia [Counseling Psychology and Psychotherapy], 27(3), 119–137. 10.17759/cpp.2019270308

[ref29] Sobkin, V.S., & Fedotova, A.V. (2019c). Teenagers in social networks: patterns of usage and aggressiveness. Journal of Siberian Federal University. Humanities and Social Sciences, 12(9), 1733–1752. 10.17516/1997-1370-0480

[ref30] Sobkin, V.S., & Skobel’tsina, K.N. (2014). Komp’iuter v zhizni rebenka-doshkol’nika [Computer in preschooler’s life]. Ditia chelovecheskoe [Human Child], 2, 20–24.

[ref31] Sobkin, V.S. (2016). Sovremennyi podrostok v sotsial’nykh setyakh [The modern teenager in social networks]. Pedagogika [Pedagogics], 8, 61–72.

[ref32] Soldatova, G.U., & Iliukhina, S.N. (2021). Autodestruktivnyi onlain-kontent: osobennosti otsenki i reagirovaniia podrostkov i molodezhi [Self-Destructive Online Content: Features of Attitude and Response of Adolescents and Youth]. Konsul’tativnaia psikhologiia i psikhoterapiia [Counseling Psychology and Psychotherapy], 29(1), 66–91. 10.17759/cpp.2021290105

[ref33] Soldatova, G.U., & L’vova, E.N. (2018). Osobennosti roditel’skoi mediatsii v situatsiiakh stolknoveniia podrostkov s onlain-riskami [Teenagers encountering online risks: characteristics of parental mediation]. Psikhologicheskaia nauka i obrazovanie [Psychological Science and Education], 23(3), 29–41. 10.17759/pse.2018230303

[ref34] Soldatova, G.U. (2018). Tsifrovaia sotsializatsiia v kul’turno-istoricheskoi paradigme: izmeniaiushchiisa rebenok v izmeniaiushchemsia mire [Digital socialization in the cultural and historical paradigm: a changing child in a changing world]. Sotsial’naia psikhologiia i obshchestvo [Social Psychology and Society], 9(3), 71–80. 10.17759/sps.2018090308

[ref35] Voiskounsky, A.E. (2010). Internet Addiction in the Context of Positive Psychology. Psychology in Russia: State of the Art, 3, 541–549.

[ref36] Vygotsky, L.S. (1984). Pedologiia podrostka [Paedology of the teenager]. In D.B. El’konin (ed.), L.S. Vygotsky. Sobranie sochinenii v 6 t. T.4. Detskaia psikhologiia [L.S. Vygotsky. Collected works: in 6 vol. Vol.4. Child Psychology] (pp. 5–242). Pedagogika.

[ref37] Walrave, M., & Heirman, W. (2011). Cyberbullying: Predicting victimization and perpetration. Children & Society, 25(1), 59–72. 10.1111/j.1099-0860.2009.00260.x

[ref38] Whittaker, E., & Kowalski, R.M. (2015). Cyberbullying Via Social Media. Journal of School Violence. Issue 1: New Directions in Cyberbullying Research, 14, 11–29.

